# Addressing the Humans in the Delivery Room—Optimising Neonatal Monitoring and Decision-Making in Transition

**DOI:** 10.3390/children12040402

**Published:** 2025-03-22

**Authors:** Christoph E. Schwarz, Bernhard Schwaberger, Alice Iride Flore, Robert Joyce, Simon Woodworth, Frederic Adam, Eugene M. Dempsey

**Affiliations:** 1 Clinic of Neonatology, Center for Pediatric and Adolescent Medicine, University of Heidelberg, 69120 Heidelberg, Germany; christoph.schwarz@med.uni-heidelberg.de; 2 INFANT Research Centre, University College Cork, T12 K8AF Cork, Ireland; s.woodworth@ucc.ie (S.W.); fadam@ucc.ie (F.A.); 3 Division of Neonatology, Department of Pediatrics and Adolescent Medicine, Medical University of Graz, 8036 Graz, Austria; bernhard.schwaberger@medunigraz.at; 4 Research Unit for Neonatal Micro- and Macrocirculation, Division of Neonatology, Department of Pediatrics and Adolescent Medicine, Medical University of Graz, 8036 Graz, Austria; 5 Department of Paediatrics & Child Health, University College Cork, T12 K8AF Cork, Irelandrobert.joyce@nmh.ie (R.J.); 6 Neonatal Medicine, Evelina London Children’s Hospital, Guy’s and St Thomas’ NHS Foundation Trust, London SE1 7EH, UK; 7 Business Information Systems, Cork University Business School, University College Cork, T12 K8AF Cork, Ireland

**Keywords:** neonatal resuscitation, delivery room, team performance, team communication, transition, newborns

## Abstract

During the first minutes of life, complex dynamic processes occur, facilitating a normal transition to ex utero life. In healthy term infants, these processes typically occur with minimal intervention required but are often more challenging for the preterm infant. These challenges involve not only the physiological processes encountered but also an organizational process: that of a team of healthcare providers led by a neonatologist, establishing a diagnosis based on clinical and technical information and initiating time-critical and potentially life-altering interventions. In this narrative review, we highlight the challenges of both processes. We explore the role and limitations of well-established and newer potential monitoring modalities, in particular respiratory function monitoring and cerebral near-infrared spectroscopy, to optimally inform the team in regards to physiological processes. We also evaluate the important role that human factors play in the process of decision-making. Both are important for optimal performance to enable successful transition and thereby reduce short- and long-term problems. We identify research goals to inform future studies to further optimize technological and human aspects in the first minutes of life.

## 1. Introduction

The first minutes of life are characterized by a significant number of interrelated dynamic physiological changes occurring simultaneously as the fetus adapts to life ex utero. Central to this process is achieving lung aeration and establishing pulmonary blood flow. Our understanding of this process has continued to evolve, mainly due to a number of important animal studies [1,2,3,4,5]. These studies are characterized by significant monitoring and invasive techniques that provide insights into the process, including lung aeration, cardiac output assessment, pulmonary blood flow and cerebral blood flow measurements, techniques that are not readily available for the newborn infant. Although the last decade has seen some advances in monitoring techniques and newer modalities of assessment are increasingly used in the delivery room (DR), objective assessment today remains characterized by pulse oximetry monitoring and electrocardiography (ECG).

In this review, we highlight some of the challenges with both of these techniques, explore the role of newer potential modalities, in particular, respiratory function monitoring and cerebral near-infrared technology, and consider the important role that human factors play in the decision-making processes that take place within care teams, as they interact amongst themselves and with the various monitoring modalities available to them in the DR. Whilst these interactions are unlikely to yield obstacles to delivery of care in the majority of cases, they become critical in those involving premature babies, who face significant challenges in establishing extra-uterine life. In these scenarios, the decision-making of the care team becomes a key element in the outcomes of care given concerns that subjectivity remains a significant factor in both diagnosis and selection of interventions [6,7] and that research suggests that it is, in fact, necessary [8], it is essential to explore every avenue to guide teams towards evidence-based decision making, analyzing both technical and non-technical factors.

## 2. Technical Factors in the Delivery Room

### 2.1. Oxygen Saturation Monitoring and Titration Strategies

Recently published results of a DR practice audit have identified oxygen titration strategies and respiratory support strategies as important areas for further improvement in neonatal resuscitation [9]. Initial starting fractionated oxygen concentrations have an important role to play in oxygen saturation targeting. Whilst the evidence is clear for term infants, it is less so for preterm infants [10,11,12]. A recent meta-analysis based on individual patient data found that high initial O_2_ (90% or higher) may be associated with reduced mortality in very preterm infants (born < 32 weeks gestation), but more evidence is required as the level of certainty was reported as low [13]. A pilot trial involving 124 infants between 28 and 33 weeks of gestation compared room air vs. 100% initial oxygen [14] identified that a longer time was required to establish regular breathing in infants initially receiving 21% O_2_. This is in line with animal model data showing that preterm rabbits receiving 21% O_2_ had lower respiratory rates and less stable breathing patterns compared to those receiving 100% O_2_ [15].

Optimal oxygenation is achieved by O_2_ saturation (SpO_2_) targeting, with adjustment of fractionated inspired O_2_ concentration to achieve the desired pre-ductal oxygen saturation at a particular timepoint during stabilization. Whilst current time-based target ranges may need to be updated to reflect delayed cord clamping strategies [16,17], key practical challenges also occur, and these need to be acknowledged. Firstly, it requires time, even in optimal situations, to obtain readings. Pulse oximetry, ECG and clinical assessment come with limitations in specific situations [18]. Some infants are bradycardic (heart rate (HR) < 100 bpm) on arrival to the resuscitaire or become bradycardic throughout the DR management period [19,20,21]. SpO_2_ is less reliable in bradycardic or in very hypoxic situations (SpO_2_ < 80%) [22,23,24]. In more active infants, movement artifacts may corrupt the signal [25,26], and its accuracy can be influenced by fetal hemoglobin [27] and low perfusion states. The provider needs to be aware of these challenges and incorporate them into their decision-making process.

The optimal oxygen titration strategy remains unclear [28,29]. Current strategies are suboptimal as infants spend a significant proportion of time outside of the target SpO_2_ ranges, predominantly hypoxia in the first minutes and hyperoxia in the latter time window [17,30]. The cause is often multifactorial: neonatal physiology, technical aspects, neonate/technology interaction, neonate/provider interaction, and provider/technology interactions. Failure to achieve a SpO_2_ of 80% at 5 min postnatally was found to be associated with adverse outcomes, including intraventricular hemorrhage in preterm neonates [31]. In a recent prospective cohort study, moderate to late preterm infants (32–36 weeks of gestation at birth) who received respiratory support often failed to achieve appropriate saturation at 5 min, with 42% having a SpO_2_ < 80% and only 19% within the target range at this time point (SpO_2_ 80–85%) [32]. Hyperoxia in the DR can generate reactive oxygen and nitrogen species [33,34] with subsequent risks of oxidative injury, including, amongst others, retinopathy of prematurity.

More frequent O_2_ titration (e.g., every 10–20 s) may improve oxygenation, but in complex scenarios, it may not be feasible for the attending physician to focus on multiple tasks simultaneously [35]. This would require an additional person acting predominantly as the ‘titrator’. The use of a visual display unit highlighting the target ranges, such as the Transitional Oxygen Targeting System (TOTS), may reduce the duration spent outside the range [36]. It may be challenging for teams to factor in the delay that occurs between adjustment at the O_2_ blender and the new concentration of O_2_ reaching the lungs, potentially leading to further delays in the infant’s HR and/or SpO_2_ response [37,38,39,40,41,42]. Therefore, automation may be one potential solution. Currently, a group from Hobart, Tasmania, is working on an algorithm for automatic oxygen control (AOC) in the early transitional phase of adaptation. The recently published pilot study found the algorithm performed comparable to manual targeting (SpO_2_ in target range (% time) 60 (48–72) during manual vs. 70 (60–84) during AOC period, *p* = 0.31) [43]. Both times within the target range appear higher than previously reported. A better understanding of the infants’ response to a fraction of inspired O_2_ adjustments might lead to improved outcomes, but this would also require improvements in monitoring, documentation, equipment, guidelines and team effort—as well as ongoing further research.

### 2.2. Heart Rate Assessment

The importance of bradycardia on preterm outcomes has been highlighted [44]. Early, accurate determination of HR is central to ongoing management. The most readily available clinical techniques include auscultation and palpation, both of which are prone to human error [45]. Simple calculations at stressful times can lead to erroneous measurements [46], resulting in either unnecessary interventions or, conversely, a lack of interventions when required. Correct application and awareness of the practical challenges in signal acquisition are key to the correct interpretation of HR, regardless of the objective method employed. Pulse oximetry readings require some time (approximately 90 s) to obtain and are prone to a number of the technical challenges highlighted previously. ECG is more accurate, especially in bradycardic situations, and faster to obtain readings compared to pulse oximetry [20,45,47,48,49,50]. The application of ECG electrodes provides the quickest determination of HR [45]. There are very few challenges to the interpretation of the ECG. The most recent guidelines provide clarity on when ECG should be used in stabilization scenarios [51].

Newer methods of HR detection are currently being evaluated [52]. These include reflectance photoplethysmography (rPPG), dry ECG electrodes, handheld Doppler, digital stethoscopes, camera photoplethysmography (PPG), capacitive sensors/electrodes, pulsatile near-infrared spectroscopy modes and laser Doppler vibrometer. Devices such as *neotap* and *neobeat* [53] have been evaluated. Each will require rigorous testing, in particular, to evaluate the human factors involved in their application, visualization, interpretation and subsequent decision-making process that arises from their use. Electrical biosensing technology could provide enhanced circulatory monitoring beyond HR alone [54]. However, further evaluation and practical issues need to be addressed prior to any potential use in the DR [55,56].

### 2.3. Potential Role of Respiratory Function Monitoring

Positive-pressure ventilation (PPV) represents the cornerstone of neonatal stabilization in the DR [57,58]. PPV should be given to any infant who is bradycardic, has no or poor respiratory effort, or remains hypoxic despite continuous positive airway pressure. Providing adequate ventilation and a reasonable tidal volume (Vt) might be technically challenging in this setting [59]. Vt is rarely measured in the DR and can vary widely, with one study finding values from 0 to 31 mL/kg during face mask PPV [60].

Typically, the adequacy of ventilation is assessed through clinical signs such as an increase in the subjective observation of chest excursion and auscultation of breath sounds [61]. However, relying on these signs may easily lead to both under and over-ventilation due to their inherent inaccuracy. Unsuccessful ventilation is frequently caused by inadvertent mask leaks and obstruction to gas flow, potentially delivering an insufficient Vt to achieve adequate gas exchange [62]. Conversely, animal studies have shown that an excessive Vt (>8 mL/kg) may cause lung injury through acute activation of different inflammatory pathways [63]. Objective quantification and feedback on Vt, mask leak and airway obstruction may improve resuscitation performance (and ultimately neonatal outcomes).

A respiratory function monitor (RFM) provides an objective assessment of ventilation through mask leak detection and Vt delivery quantification [64]. Several different devices exist, but each typically provides a graphical display of Vt, airway pressure and leak. Their efficacy has been demonstrated in several manikin studies [65,66,67]. Clinician performance improves during simulated resuscitation when this is guided by continuous feedback on ventilation [68]. However, translating these into enhanced respiratory management in the DR has not been proven. To date, three randomized controlled trials have been performed [69,70,71]. A feasibility study compared the use of an RFM to improve mask ventilation versus standard clinical assessment in a cohort of 49 preterm infants receiving PPV in the DR [69] and observed a reduced mask leak in the RFM visible group in comparison to the masked group (37 [22–54]% vs. 54 [37–82]%, *p* = 0.01), as well as more mask repositioning and positive inspiratory pressure adjustments. Although median Vt was similar, a higher Vt (>8 mL/kg) was observed less frequently in the RFM visible group (0.81; 95% CI, 0.67–0.98). There were no significant differences in intubation rate in the first 24 h, death or grade 4 intraventricular hemorrhage and death before discharge. A study of preterm infants by Zeballos et al. reported significantly higher Vt in the RFM masked group compared with the RFM visible group (7 [5.7–9.2] vs. 5.8 [4.7–6.8] mL/kg; *p* < 0.001), and fewer infants with an excessive Vt (>8 mL/kg) in the RFM group. However, no differences in clinical outcome measures, such as surfactant use or intubation rate, were found [71]. The last study, the multicenter MoNitoR trial, randomized preterm infants to either a visible or non-visible RFM during stabilization. Despite the multicenter nature of the study and the investigation of over 50,000 inflations, the authors did not find any difference between the two groups in the percentage of inflations delivered within a specific expired Vt target range (Vt 4–8 mL/kg). The rate of intraventricular hemorrhage and/or cystic periventricular leukomalacia was found to be lower in the RFM-visible group despite no actual difference in minute ventilation, Vt, or other RFM measurements [72]. A recent meta-analysis including 443 infants showed no reduction of risk of death with the use of a visible RFM versus no RFM in the DR. However, providing PPV with an RFM may result in a reduction in brain injury [73].

The use of the RFM highlights many of the challenges in incorporating new practices into clinical care. A RFM requires appropriate formal training prior to its clinical use since inexperience may lead to misinterpretation of the signals and inadequate/incorrect decision-making. Visual assessment of RFM waveforms has shown poor interrater reliability in a small observational study, questioning the generalisability of results in this research field [74]. In a recent survey sent to 106 clinicians involved in RFM-guided clinical trials, 78% felt that they would benefit from additional training [75]. Most respondents considered the RFM helpful to guide neonatal ventilation and help decision-making. Another suggested improvement is the need to adopt a simpler visual interface, enabling easier information processing in the stressful context of resuscitation. It has been postulated that the use of this additional tool may deviate clinicians’ attention from the baby to the monitor, an observation that has been made in other research studies involving the use of information systems or computer applications implemented at a point of interaction between a patient and a clinician. In one study, a nurse made the point that it was the patient that she wanted to look at, not a monitor or keyboard [76]. In an ancillary study of the MoNitoR trial, visual attention during neonatal resuscitation was quantitatively studied with eye-tracking [77]. The presence of an RFM was associated with altered providers’ visual attention, with increased total gaze duration spent on the device. These results highlight the need for a more extensive insight into human attention in simulated and real clinical scenarios.

Thus, PPV remains one of the least controlled interventions in the DR. A RFM has been demonstrated to be a safe way to reduce mask leak and reduce excessive Vt in manikin studies and small observational studies, although randomized clinical trials have produced inconsistent results. Whilst the available evidence does not currently support the routine use of RFM in the setting of the DR studies, addressing the human factor aspects of interaction with the device is warranted.

### 2.4. Potential Role of Cerebral Oxygenation Monitoring

Advanced monitoring methods to shift the focus to the most vulnerable organ, the premature brain, are a growing area of interest. In recent years, near-infrared spectroscopy (NIRS) has been increasingly employed for assessing cerebral oxygenation and perfusion in neonates [78,79,80]. Various NIRS monitors have been introduced for clinical use, and reference ranges are derived from healthy late-preterm and full-term neonates [81,82,83]. Studies have shown correlations between reduced cerebral tissue oxygenation in the first minutes after birth and the occurrence of intraventricular hemorrhage [84,85], as well as short-term [86] and long-term neurological outcomes [87]. Based on these findings, cerebral NIRS monitoring may facilitate the identification of neonates in need of additional medical interventions, such as optimizing oxygen delivery and respiratory support, potentially improving outcomes in this high-risk population. The COSGOD (phase I/II) pilot feasibility trial [88], conducted in premature neonates born at less than 34 weeks gestation, demonstrated a reduction in cerebral hypoxia within the first 15 min after birth. Following a similar approach, the most recent multicenter randomized controlled COSGOD III trial [89] showed a 4.3% increase in survival without cerebral injury in premature neonates born at less than 32 weeks gestation through the use of cerebral NIRS monitoring, although these results did not reach statistical significance. From the COSGOD III cohort, centiles for cerebral tissue oxygenation during postnatal stabilization in extremely (<28 weeks) and very premature neonates (<32 weeks) with favorable outcomes were established [16]. These centiles may serve as reference ranges for guiding medical interventions during the stabilization and resuscitation of premature neonates in the future. When implementing cerebral NIRS monitoring into clinical practice, one challenge may lie in integrating these additional parameters and their dynamic references into the monitoring systems. This may help healthcare providers perceive and respond appropriately to these additional signals while accounting for such human factors as cognitive load, stress, and reaction time. Limitations such as signal variability, sensor displacement, and the influence of systemic hemodynamic factors on NIRS readings must be considered, as these can impact measurement accuracy and clinical interpretation.

### 2.5. Potential Role of Video Laryngoscopy

The use of video laryngoscopy (VL) is evolving in neonatal care. A recent Cochrane review including 8 studies identified its potential benefits in increasing the rate of intubation at first attempt while reducing airway-related adverse effects [90]. A recently published single-center RCT evaluating emergency intubation attempts in neonates supported VL use: Successful first-attempt intubations occurred in 74% of the VL group compared to 45% in the conventional laryngoscopy group (*p* < 0.001) [91]. However, in this trial, intubation attempts were mainly performed in the NICU (71%) and not in the DR (29%), where data remains limited. Other than its potential benefits on technical success, VL would appear to have a positive impact on many human factor aspects, including reducing the authority gradient among team members and potentially improving patient safety [92]. Anesthetic assistants reported improved ability to anticipate the next step and enhance teamwork in a recent survey study of VL use [93]. It is likely we will continue to see an increased use of the DR in the future.

## 3. Human Factors and Decision-Making in the Delivery Room

It is increasingly recognized that “human factors” have a significant impact on the outcomes of resuscitations. This is because the type of scenario that is prevalent in the DR, similar to other scenarios involving extreme or critical decision-making, places significant pressure on decision-makers [94]. The workload, the multiple cognitive challenges, the requirement for high degrees of expertise, and the communication intensity will present difficulties that make identifying a diagnosis and appropriate interventions far more difficult. In settings where only a small number of such cases occur, perhaps less than one per month, the complexity of care may prove insurmountable for less experienced individuals, and this is a concern.

The non-technical, human factors that determine the performance of teams in these extreme circumstances, such as leadership, behavior, and communication, have traditionally been viewed as ‘intangible’ factors inherent in individuals. It was considered that those leading resuscitations were either good communicators or they were not, could lead a team effectively or could not. More recent training methods, however, have demonstrated that leadership and other non-technical skills can be developed and taught; programs incorporating resuscitation simulations and associated boosters have been shown to increase non-technical skills scores in participants [95,96,97,98].

### 3.1. Team Composition and Team Performance

In other domains, the composition of teams as an avenue for optimizing their performance had been noted early on, and definitive propositions had been made towards improving their performance, particularly in situations involving high-pressure decision-making. The seminal work of Torrance in the 1940s and 1950s is probably one of the earliest examples of this direction of research. Torrance studied the dynamics of the crew members’ behavior in the military, notably bomber aircraft crews and ascertained that their performance during difficult missions in enemy territory and in survival conditions could be improved or lessened by certain attributes and behavioral factors [99].

The personality of the group leader and their behavior came to particular attention, where their ability to keep briefing other members of the crew during the mission had been found to greatly help them to keep calm in the face of danger [100]. On the contrary, the feeling of helplessness that was associated with crew members not having frequent access to information was found to destroy the overall performance of the team. This seems consistent with Torrance’s base observation that “a group of skilled individuals does not necessarily make an effective group” [101] and that, to improve group performance, it is imperative to identify what is wrong with it in the first instance. These observations seem to bear some relevance to our inquiry about the performance of neonatal teams in the DR, where a team of doctors and nurses with various levels of expertise must collaborate to deliver the best possible outcome. Certainly, the ability of the leader in the room to communicate effectively with their “crew” seems very pertinent. In Torrance’s studies, the emotions displayed by the person in charge were also determinant in channeling other crew members’ emotions, with expressions of uncertainty by leaders being found to be very contagious for other crew members. It would be valuable to use video analysis to determine whether these findings apply to teams working in the DR.

Clarity regarding roles within a team environment allows for an increase in efficiency and improvement in overall output. Learnings from other professions (e.g., aviation, sports) can be utilized to define responsibilities, streamline processes, and construct checklists. In preterm infants, where timely management is particularly critical, this has the potential to improve staff confidence and patient outcomes [102]. Deliberate strategies that seek to define and develop non-technical skills are required in clinical settings and should be considered aspects of the simulation framework for neonatal resuscitation. Rather than considering technical and non-technical skills as independent variables influencing the “success” of resuscitation, non-technical skills should be considered a platform utilized to increase the efficacy of technical performance and to optimize resuscitation compliance.

Communication within the team has been identified as critical, and within an established framework, it is certainly a factor of performance that responds well to training, especially training by way of simulation. Bavelas had proposed experimental protocols to test the most effective patterns of communication [103]. His experiments, which have been extended since, notably by Lewin [104] and Baron and Byrne [105], indicated that the type of problems faced by decision-making teams had a significant influence on their problem-solving performance. For complex problems, groups that relied markedly on their leader could become slower if it led to a cognitive overload of that individual, such that more open groups could be faster if the task was shared more equally amongst team members. This could be an interesting factor to observe in the DR, where the senior neonatologist naturally plays the role of the leader, and the extent to which all actions and interactions pass through them could lead to less effective decisions. Given the importance of timely intervention in the DR, this is a critical area of concern.

### 3.2. Methodological Caveats in Understanding Human Factors in Decision-Making

However, defining with precision the attributes and behaviors that make a very effective resuscitation team will be very challenging [106]. A survey of healthcare providers attending neonatal resuscitations identified factors such as leadership, team composition, and communication as essential aspects of effective teamwork in resuscitation [106,107]. These aspects are observational and perceptible in nature and can be incorporated into simulation training with feedback provided to the trainees [108]. Torrance’s approach to his research is also relevant here, where he used a combination of review work and an investigation of over 1000 reported incidents amongst his target groups of 200 crew members who had survived missions in World War II and the Korean War. This led to a group test protocol and training program, which were subsequently proposed to the US Air Force [99,100].

Technical and non-technical skills are naturally interlinked and influence overall team performance. Videos of neonatal resuscitations have demonstrated that teams with high scores in non-technical skills also score highly in overall performance in terms of adherence to resuscitation guidelines [109,110]. Simulation training that incorporates aspects of teamwork has been shown to increase overall scores in resuscitation scenarios amongst neonatal healthcare workers [111]. One added difficulty in the interpretation of recordings of actual interventions in DRs comes from the fact that, in the real world, all these technical and non-technical factors arise and interact all at once, blurring the cause-and-effect relationship between them and making the identification of their individual impact uncertain. This is where simulation-based studies may provide opportunities to stage scenarios where specific factors are at play, making the analysis of their impact easier. This proposal comes with one caveat: in other areas of study of human decision-making, the reliance on experimental protocols and simulations has shown its limitations where participants’ behavior in simulations deviated from how they would behave in real cases. For instance, the validity of experimental research in jury behavior in criminal cases was reviewed in detail by Curley and Peddie, and they propose different measures of validity of this research as it relates to the reality of actual jury trials [112]. Of particular note is their observation that there is a trade-off between designing experiments that are very realistic (and difficult to interpret, therefore) and designing simple experiments that are easy to interpret but have a low relevance in understanding real-life interventions.

### 3.3. The Decision Making Process

One key aspect of the work of a resuscitation team is, of course, the diagnosis and the therapeutic decision-making that follows. Human decision-making has been the focus of much attention and research for quite some time. Herbert Simon and his work on rationality, for which he won the Nobel Prize in Economics in 1978, offers a robust model on which to base any investigation of human decision-making as a reasoned endeavor [113]. However, notwithstanding the importance of Simon’s work, Gary Klein’s work on the decision-making processes of emergency responders (nurses and firemen) seems particularly relevant to any investigation of clinical decision-making, particularly that which occurs in the DR. In relative contrast to Simon’s theoretical work, Gary Klein promoted a mode of research on decision-making, which he labeled Naturalistic, meaning that it was based on the observation of expert decision-makers rather than on a normative view of how human agents should make decisions (cf: Simon’s 1947 process of decision making). Naturalistic decision-making delivered some interesting empirical results, which he formalized as Recognition-Primed Decision Making (RPDM) [114]. His analysis focuses on the expertise of the decision maker and their ability to (1) recognize patterns of events previously encountered to apply them to new cases and (2) monitor the impact of their interventions to validate their diagnosis. RPDM is particularly relevant in scenarios where time pressure is high, and the correctness of decisions is critical.

Klein’s work is particularly interesting because it posits the key difference between novice decision-makers and expert decision-makers. Whilst we have already considered that there are different levels of expertise in resuscitation teams (e.g., typically a senior and a junior doctor), we can consider that such teams are expert teams led by an expert neonatologist. This is a scenario where RPDM is likely to provide the best fit for explaining patterns of decision-making as they occur in real-life interventions. Expert decision makers, having access to a broad set of retrospective cases that they have attended to and have memorized, are able to use recognition as a tool for diagnosis and implementing solutions. Solutions that have proven successful in the past in cases that resemble the current case will come to the fore and become candidate solutions. As these solutions are implemented by the care team, the expert neonatologist will evaluate the patient’s response particularly closely and track whether it is aligned with the past case they felt they recognized. Deviations from the expected scenario will drive them to abandon their diagnosis and pursue a different avenue, corresponding to another case that seems to fit. Obviously, the more relevant experience a neonatologist will possess, the more diverse the set of retrospective cases they can recall will be. It is interesting to consider how the profile of patients coming through a particular facility will influence the speed at which expertise can be built up. This is particularly important for certain categories of cases, notably very early premature babies.

At stake in the discussion of how experts make decisions is one factor of particular importance in the DR: speed of decision-making. Expert decision-makers make faster decisions, which, when time frames become impossibly short, do not rely on reasoning but leverage a portion of human cognition that is very difficult to observe formally: intuition. Dane and Pratt [104] explain how intuition is different from guessing or “betting” and relies on accumulated expertise and experience, using undetectable processes known as “bunching” of knowledge [115]. Bunching entails creating mental associations between factors (e.g., between causes and effects), which are used to underpin decision-making in often unconscious ways that take place within seconds. When asked about intuition, decision-makers describe how they can achieve great confidence that their decisions are correct nearly instantaneously. Whilst the ability of experts to make rapid and precise decisions appears to be an advantage in very acute situations—indeed, it has often been celebrated in the literature on decision-making [116], it is unsatisfactory from regulatory and governance viewpoints where the evidence-based nature of decision making is a requirement. Because intuition is not auditable, there are aspects to RPDM-based decision-making scenarios that are not viable in the DR in the absence of strict protocols and without an array of information systems to guide it. It has led authors to characterize the reliance on “gut feeling” or intuition as inherently dangerous, even in business contexts [117]. Thus, there is a lasting debate on whether “fast and frugal” decision-making [118,119] is the optimal way to tackle situations that require rapid decisions to be made.

Future research must concentrate on exploring how decisions are made in delivery rooms, using such comprehensive data collection methods as video recordings, in order to present a coherent picture of the clinical science leveraged by experts, the current and potentially new information systems utilized in the delivery room and the human factors at play within clinical teams. We can then consider how these three elements combine to deliver a safe and effective environment for decision-making in the DR, promoting rapid response while at the same time also promoting objectivity and evidence-based decision-making.

## 4. Conclusions

The first minutes of life represent a convergence of two complex processes: a biological one, as the baby transitions from intra-uterine to extra-uterine life, and an organizational one, as a team of healthcare providers led by a neonatologist establishing a diagnosis and implementing potentially life-altering interventions. This paper has explored the dimensions that contribute to determining the outcome of this convergence: a positive outcome where a patient makes a successful transition without potential long-term adverse health impacts. These dimensions come from different domains: the science of neonatal resuscitation, the technology derived from this science, and the human factors that are involved in the performance of a team of experts. Our review indicates that much is still unknown about how the processes to be implemented in the DR can be optimized, with science informing the tools and practices used by neonatologists. Future research must concentrate on proposing a specific agenda that is structured around blocks of knowledge and practice that can help us organize our research endeavors in a coherent manner. The accompanying Table 1 proposes a synthesis of our proposals for future avenues of research, which must coalesce into a coherent strategy for amending the approach to neonatal care in the DR. Therefore, new interventions can emerge that may improve the health outcomes of the most at-risk patients.

## Figures and Tables

**Table 1:** Synthesis of proposals for a future research agenda.

Proposals for Future Research
Experiment with embedding new tools and new measurements pertaining to oxygen monitoring, heart rate assessment, respiratory function monitoring and cerebral oxygenation monitoring in the DR Explore the visualization, interpretation and subsequent impact on decision-making that arises from their use Explore the broader impact of their implementation on the process and outcomes of care Integrate new tools and techniques into the overall patient monitoring systems to help healthcare providers perceive and respond appropriately to these additional signals, even in the high-stress context of neonatal emergency situations in the DR Explore the possible impact on efficacy and speed of decision-making of analysis, modeling, synthesis and abstraction of multiple data streams Explore the efficacy of feedback and control loops (human-mediated or otherwise) in stabilizing oxygenation Explore the applicability of decision space theories in formulating relationships between physiological data, team efficacy and patient outcomes Develop specific training procedures that ensure that operators understand how to apply, interpret and analyze the data provided by the new instruments Understand the importance of human factors, particularly non-technical factors, on the performance of DR teams Measure the occurrence of stress and the impact of extreme time pressures in the DR Investigate the importance of the composition of the DR team Explore the importance of the personality and communication abilities of the leader Explore opportunities offered by newly developed training protocols for non-technical skills Explore the importance of leveraging the expertise and experience of team members and the role of intuition in accelerating diagnosis and decision-making Investigate whether fast and frugal decision-making takes place in the DR Apply the latest video recording equipment to generate high-quality multi-orientation video analysis of team movement and communication in the DR Design simulation/experiments that associate both realism and ethical considerations Bring to bear three fundamental bodies of knowledge: (1) the medical science of neonatology, (2) technologies and techniques that are imported from other domains and (3) team dynamics and decision-making processes to deliver a safe and effective environment for decision-making in the DR which promotes rapid response, to the same extent as it promotes objectivity and evidence-based decision making

## Data Availability

Not applicable.

## Abbreviations

The following abbreviations are used in this manuscript:

AOC	Automatic oxygen control
DR	Delivery Room
ECG	Electrocardiography
HR	Heart rate
NICU	Neonatal intensive care unit
NIRS	Near-infrared spectroscopy
O_2_	Oxygen
PPV	Positive-pressure ventilation
RFM	Respiratory function monitor
RPDM	Recognition-primed decision making
SpO_2_	pulse oximetry measured hemoglobin O_2_ saturation
TOTS	Transitional Oxygen Targeting System
VL	Video laryngoscopy
Vt	Tidal volume
